# Molecular Epidemiology of TGF‐β1 Gene Variants and Their Relationship With Adult Type 2 Diabetes Mellitus Patients Visiting Injibara Comprehensive Hospital in Northwest Ethiopia: A Case‐Control Study

**DOI:** 10.1002/hsr2.73298

**Published:** 2026-09-25

**Authors:** Haymanot Getnet, Mequanente Dagnaw, Meera Indracanti, Nega Berhane

**Affiliations:** ^1^ Department of Medical Biotechnology, Institute of Biotechnology University of Gondar Gondar Ethiopia; ^2^ Department of Epidemiology and Biostatistics, Institute of Public Health University of Gondar Gondar Ethiopia; ^3^ Department of Medical Biotechnology School of Allied and Healthcare Sciences, Malla Reddy University Hyderabad Telangana India

**Keywords:** ARMS PCR techniques, risk of type 2 diabetes mellitus, T2 DM, TGF‐β1 gene polymorphism

## Abstract

**Background and Aim:**

Type 2 diabetes mellitus (T2DM) is a chronic metabolic disorder influenced by environmental and genetic factors. The TGF‐β1 codon 10 T > C polymorphism has been investigated as a potential genetic factor for T2DM, but evidence from African populations, particularly Ethiopia, remains limited. This study aimed to assess the association between TGF‐β1 codon 10 T > C polymorphism and T2DM among patients attending Injibara Comprehensive Hospital, Northwest Ethiopia.

**Methods:**

A case‐control study was conducted among 230 participants (115 T2DM cases and 115 controls). Sociodemographic, behavioral, and clinical data were collected using a structured questionnaire. DNA was extracted from blood samples, and TGF‐β1 codon 10 T > C polymorphism was genotyped using ARMS‐PCR. Associations were assessed using bivariate and multivariable logistic regression.

**Results:**

The TC genotype was significantly associated with higher odds of T2DM compared with the TT genotype (AOR = 3.326, 95% CI: 1.009–10.969, *p* = 0.048). The C allele was also associated with higher odds of T2DM (AOR = 2.361, 95% CI: 1.231–4.528, *p* = 0.010). However, the CC genotype was not significantly associated with T2DM (*p* = 0.739). Overweight BMI, elevated blood pressure, and alcohol consumption were also significantly associated with T2DM.

**Conclusion:**

The TGF‐β1 codon 10 TC genotype and C allele were associated with T2DM in this study, whereas the CC genotype was not significantly associated. These findings should be interpreted cautiously and require confirmation in larger studies.

## 1 Introduction

Type 2 diabetes mellitus (T2DM) is a rapidly increasing non‐communicable disease and a major public health challenge, particularly in low‐ and middle‐income countries such as Ethiopia. It is characterized by chronic hyperglycemia caused by insulin resistance and impaired insulin secretion. Its increasing burden is linked to urbanization, sedentary lifestyles, obesity, unhealthy diets, and population aging, with substantial morbidity, mortality, and healthcare costs. T2DM can also cause serious complications, including cardiovascular disease, nephropathy, neuropathy, and retinopathy [1, 2].

Genetic susceptibility, in addition to environmental and behavioral factors, contributes to T2DM. TGF‐β1 is a potential candidate gene because of its roles in inflammation, immune regulation, fibrosis, insulin resistance, and pancreatic β‐cell function. Variations in TGF‐β1 expression may therefore influence T2DM development and complications, and several studies have reported associations between TGF‐β1 polymorphisms and T2DM in different populations [3, 4].

Although previous studies have demonstrated associations between TGF‐β1 gene variants and T2DM, findings remain inconsistent across ethnic groups and geographic regions. Some studies identified significant associations between the T/C genotype and increased risk of T2DM, whereas others reported weak or no association [5, 6]. These discrepancies may be attributed to differences in genetic background, environmental exposure, sample size, and study design. Moreover, most molecular epidemiological studies on TGF‐β1 polymorphisms have been conducted in Asian and European populations, while evidence from African populations, particularly Ethiopia, remains very limited.

Current T2DM management emphasizes lifestyle modification, early screening, glycemic control, and prevention of complications. Genetic research may improve the identification of individuals at increased risk and inform personalized approaches. However, evidence on TGF‐β1 polymorphisms and T2DM in Ethiopian populations remains limited. Therefore, this study assessed the association between TGF‐β1 codon 10 T > C polymorphism and T2DM among patients attending Injibara Comprehensive Hospital in Northwest Ethiopia.

## 2 Methods

### 2.1 Study Area

The study was conducted at Injibara Comprehensive Hospital in Injibara town, Amhara Region, Northwest Ethiopia. The hospital provides emergency, outpatient, inpatient, and surgical services and serves over 1.32 million people in the Awi Zone [7].

### 2.2 Study Design and Period

A case‐control study was carried out from February, 2024–January 2025.

### 2.3 Population

#### 2.3.1 Source Population

The source population was all patients with diabetes diagnosed by physicians according to WHO criteria [8] during the study period.

#### 2.3.2 Study Population

All T2DM patients (aged ≥ 18 years) diagnosed by physicians participated. The healthy volunteers as controls age, who were available during the study period and were able and willing to give informed consent.

### 2.4 Inclusion Criteria and Exclusion Criteria

Inclusion criteria: adults (≥ 18 years) with confirmed T2DM who provided informed consent, and age‐matched healthy controls willing to participate and provide informed consent.

Exclusion criteria: participants with conditions that could affect the results, including active infection, cancer, autoimmune diseases, or those who declined participation.

### 2.5 Sample Size Determination and Sampling Technique

Because the primary objective was to assess the association between TGF‐β1 T869C polymorphism and T2DM, an unmatched case‐control sample size formula was used. Based on a control C‐allele frequency of 18%, an expected OR of 2.36, 5% significance level, 80% power, and a 1:1 case‐control ratio, the required sample was 115 participants per group (230 total).

Adults with confirmed T2DM were consecutively recruited as cases, while apparently healthy adults without T2DM attending the same hospital were consecutively recruited as controls. Thus, 115 cases and 115 controls were enrolled after eligibility confirmation and written informed consent.

### 2.6 Variables

#### 2.6.1 Dependent Variable

T2DM: this is the primary condition being studied, specifically its association with TGF‐β1 gene polymorphism among patients.

#### 2.6.2 Independent Variables

TGF‐β1 gene polymorphism: genetic variation in the TGF‐β1 gene potentially associated with T2DM.

Associated factors: demographic, behavioral, and clinical factors potentially associated with T2DM.

### 2.7 Operational Definition

TGF‐β1 gene polymorphism: Genetic variation in the TGF‐β1 gene that may affect its expression and function. In this study, the TC genotype and C allele were associated with higher odds of T2DM, whereas the CC genotype was not significantly associated with T2DM [9]. These findings suggest that the TC genotype and C allele may be associated with higher odds of T2DM. However, the CC genotype was not significantly associated with T2DM; therefore, causal or protective effects should not be inferred [10]. The allelic frequency of these polymorphisms was assessed in both T2 DM patients and healthy controls.

T2DM: T2DM is a chronic metabolic disorder characterized by insulin resistance and relative insulin deficiency, resulting in elevated blood glucose levels [11, 12]. In this study, T2DM is defined operationally as patients who have been clinically diagnosed with the condition and are currently receiving follow‐up care at the hospital.

Healthy controls: Individuals without T2DM or related metabolic disorders, matched to cases by age, sex, and relevant demographic factors, and used as the comparison group for assessing the association between TGF‐β1 polymorphisms and T2DM.

Associated factors of Type 2 DM: This encompasses various demographic, clinical, and lifestyle factors that may contribute to the risk of developing T2DM among the study populations. Factors may include age, body mass index (BMI), family history of diabetes, dietary habits, physical activity levels, smoking, and other health conditions [13].

### 2.8 Data Collection

The socio‐demographic, clinical, and behavioral characteristics of the patients, such as gender, age, duration of diabetes, family history, smoking, BMI, fasting glucose test, and others, were recorded using a semi‐structured questionnaire (Anex).

Three milliliters of the blood samples were collected from all subjects of diabetic patients and controls. The blood was kept in an Ethylene Diamine Tetra Acetic Acid (EDTA) tube for isolation of DNA and stored at −22°C.

### 2.9 Laboratory Method

#### 2.9.1 Genomic DNA

Extraction process using the modified salting‐out method in the previous study [14]: Genomic DNA was extracted from blood samples using the salting‐out method. Those methods include: reagent preparation, red blood cell lysis, cell lysis, precipitation of DNA, and preservation at −22°C for the next work according to the protocol [14].

#### 2.9.2 The Quality and Quantity of the Isolated DNA

DNA concentration and purity were assessed using a NanoDrop spectrophotometer and gel electrophoresis. DNA purity was evaluated using the A260/A280 ratio, with higher ratios indicating greater purity [14]. DNA purity was assessed using a NanoDrop, with an A260/A280 ratio of 1.8–2.0 considered acceptable. DNA integrity was confirmed by gel electrophoresis and documented using a gel documentation system [15]. ARMS‐PCR primers were used for allele‐specific detection of the TGF‐β1 codon 10 T > C polymorphism, following a previously published method [16]. PCR primer sequences of TGF‐β1 codon 10. In this case, two allele‐specific primers (R1 and F2) and two common primers (F1 and R2) are specified for the TGF‐b1 gene (Table S1). F1 and R2 outer primers have a base pair product of 465 bp, R1 C allele‐specific and F2 T allele‐specific base pairs of 204 and 297, respectively. These primers were designed using SnapGene Viewer software, depending on the previous study information [16, 17], to amplify specific regions of the *TGF‐β1* codon 10 T > C gene. Resulting in PCR products of different lengths (204 bp for R1, 297 bp for F2, and 465 bp for F1 and R2). Therefore the PCR reactions were optimized for a 20 μL final volume using a 10 μL mix, 2 μL of genomic DNA,0.5 µL of each primer (four different primers), and completed with nuclease‐free water [16].

PCR was performed with an initial denaturation at 95°C for 5 min, followed by 30 cycles of 95°C for 30 s, 67°C for 40 s, and 72°C for 1 min, with a final extension at 72°C for 7 min. All four primers were used in a single reaction to detect the wild‐type (T) and mutant‐type (C) alleles [18].

#### 2.9.3 Agarose Gel Electrophoresis

A 100‐bp DNA ladder was used as a size marker. PCR products were separated by electrophoresis on a 2% agarose gel in 1× TBE buffer at 100 V for 45–60 min, stained with ethidium bromide, and visualized using a gel documentation system [16].

### 2.10 Data Quality

Data quality was ensured through pretesting, translation, and back‐translation of the questionnaire, appropriate revisions, and daily checks of data completeness by the supervisor and principal investigator.

### 2.11 Statistical Analysis

Statistical analyses were performed using SPSS version 25. Frequencies, percentages, *χ* ^2^ tests, and binary logistic regression were used. Variables with *p *< 0.25 in bivariate analysis and clinically relevant covariates were included in multivariable analysis. Adjusted odds ratios (AORs) with 95% CIs were reported, with *p* < 0.05 considered statistically significant. Hardy‐Weinberg equilibrium (HWE) was assessed separately in the control group using the observed genotype frequencies. Allele frequencies were calculated as *p* = *f*(T)+ ½*f*(TC) and *q* = *f*(C)+ ½*f*(TC), where *p* and *q* represent the T and C allele frequencies, respectively, and *p* + *q* = 1. Expected genotype frequencies were calculated as, TT = *p*2, TC = 2*pq*, and CC = *q*2. The observed and expected genotype frequencies among controls were compared using the*χ* ^2^ goodness‐of‐fit test. A *p*‐value > 0.05 was considered consistent with HWE. The HWE results for the control group, including the observed and expected genotype frequencies, statistic, and *p* value, are reported separately in Section Results, 3 and should not be confused with the *χ* ^2^ test comparing genotype distributions between cases and controls.

## 3 Results

### 3.1 Socio‐Demographic Parameters of Study Participants

Among 230 participants (115 cases and 115 controls), 51.7% were male, and 61.7% were aged 41–60 years. Most were married (80.0%), had non‐formal education (51.3%), and earned > 6000 ETB/month (73.9%). Businessmen/dealers and farmers were the most common occupations, and 97.0% were Orthodox Christians (Table S2).

### 3.2 Behavioral and Clinical Parameters of Study Participants

The study compares lifestyle behaviors and clinical parameters between two groups (cases and controls, each with 115 individuals; total = 230). Results are presented as frequency and percentage, highlighting distinct patterns in both behavioral and clinical variables across the groups (Table S3).

### 3.3 Genetic Perimeters of the Study Participants

ARMS‐PCR genotyping identified three TGF‐β1 codon 10 genotypes (TT, TC, and CC). The 297‐bp band indicated the T allele, the 204‐bp band indicated the C allele, and the 465‐bp band represented the common outer primer control (Figures S1 and S2).

### 3.4 GF‐B1 Genotype and Allelic Distribution

A significant difference was observed in TGF‐β1 codon 10 genotype distribution between T2DM cases and controls (*χ* ^2 ^= 18.019, *p* < 0.001). The TC genotype and C allele were more frequent among cases than controls. Among controls, genotype frequencies were consistent with Hardy–Weinberg equilibrium (*χ* ^2 ^= 0.53, *p*= 0.47) (Table 1).

**Table 1:** Distribution of *TGF‐β1* codon 10 genotypes and allele frequencies among T2DM patients and controls.

Group	TT, *n* (%)	TC, *n* (%)	CC, *n* (%)	T allele, *n* (%)	C allele, *n* (%)	*χ* ^2^	*p* value
T2DM	46 (40.0)	58 (50.4)	11 (9.6)	150 (65.0)	80 (35.0)	18.019	< 0.001
Control	78 (67.8)	32 (27.8)	5 (4.3)	188 (81.7)	42 (18.3)		
Total	124 (53.9)	90 (39.1)	16 (7.0)	338 (73.5)	122 (26.5)		

### 3.5 Bivariate and Multivariate Analysis of Associated Factors With Type 2 DM

The association between selected socio‐demographic and clinical factors with T2DM was assessed using bivariate and multivariable logistic regression analyses. In the bivariate analysis, occupation status was significantly associated with T2DM. Compared with housewives, farmers were 68.3% less likely to have T2DM (COR = 0.317, 95% CI: 0.136–0.741, *p*= 0.008), and daily laborers were 83.3% less likely to develop T2DM (COR = 0.167, 95% CI: 0.029–0.953, *p*= 0.044). Although businessmen showed lower odds of T2DM (COR = 0.543, 95% CI: 0.244–1.205), the association was not statistically significant (*p *= 0.133). Similarly, sex, age group, educational status, marital status, place of residence, religion, family history of diabetes mellitus, family history of hypertension, and average monthly income did not show statistically significant associations with T2DM in the crude analysis (*p *> 0.05) (Tables S4 and S5).

The TT genotype was more common among controls (67.8%) than cases (40%) and served as the reference. The TC genotype was significantly associated with higher odds of T2DM in both crude (COR = 3.730, 95% CI: 1.219–11.412, *p* = 0.021) and adjusted analyses (AOR = 3.326, 95% CI: 1.009–10.969, *p* = 0.048). The CC genotype was not significantly associated with T2DM (*p* = 0.739).

Allelic analysis showed that the C allele was more frequent among cases (35%) than controls (18%) and was associated with higher odds of T2DM (AOR = 2.361, 95% CI: 1.231–4.528, *p*= 0.010). The TC genotype was also associated with T2DM. These findings indicate an association, but not causation (Table S6).

No significant associations were observed between TGF‐β1 codon 10 T > C polymorphism and the assessed sociodemographic, behavioral, or clinical factors, including BMI, blood pressure, and alcohol consumption (all *p*> 0.05). This suggests that these factors were not significantly associated with TGF‐β1 genotype distribution in this study (Table S7).

## 4 Discussion

T2DM is influenced by genetic, environmental, and metabolic factors. TGF‐β1 is a multifunctional cytokine involved in inflammation, immune regulation, extracellular‐matrix remodeling, and fibrosis. The TGF‐β1 T869C polymorphism (rs1800470; +869 T > C) causes a Leu10Pro substitution in the signal peptide, potentially affecting TGF‐β1 production and secretion and thereby contributing to diabetes‐related processes [19, 20].

In this study, TGF‐β1 T869C genotype distributions differed significantly between T2DM cases and controls. The TT genotype was more common among controls, while the TC genotype and C allele were more frequent among cases. After adjustment, the TC genotype (AOR = 3.326, 95% CI: 1.009–10.969) and C allele (AOR = 2.361, 95% CI: 1.231–4.528) were associated with higher odds of T2DM. However, these findings indicate association rather than causation.

The observed association has biological plausibility. The T869C variant changes leucine to proline at position 10 of the TGF‐β1 signal peptide, and functional evidence indicates that the Pro10 variant can influence TGF‐β1 secretion. A study demonstrated that the Pro10 variant was associated with increased TGF‐β1 secretion in vitro [20]. Similarly, reported that the TGF‐β1 Pro10 (C) allele was associated with higher TGF‐β1 secretion and discussed its possible relevance to T2DM [9]. Thus, altered TGF‐β1 production could provide a potential biological pathway linking this polymorphism with metabolic and inflammatory processes. However, circulating TGF‐β1 concentrations were not measured in the present study; therefore, this proposed mechanism remains hypothetical.

Our findings are consistent with evidence from some populations. A study of Egyptian patients reported higher frequencies of the C allele and TC genotype among individuals with T2DM [9]. Similarly, a study from North India reported that the 869C allele and CC genotype were associated with T2DM in two population samples [21]. These findings provide support for a possible association between TGF‐β1 T869C and T2DM in populations geographically closer to Ethiopia. Nevertheless, genetic associations can vary across populations because of differences in ethnic background, allele frequencies, environmental exposures, sample size, and linkage with other genetic variants.

Evidence from pooled analyses also indicates that the association is not completely consistent. A meta‐analysis of six case‐control studies found that the CC genotype was associated with increased T2DM risk (OR = 1.397, 95% CI: 1.041–1.874), whereas the C‐versus‐T allele comparison was not statistically significant [22]. This distinction is important because it suggests that the relationship may depend on the genetic model used. Therefore, the association observed in the current study should be considered preliminary and requires confirmation in larger Ethiopian populations.

No significant associations were observed between TGF‐β1 T869C genotypes and the examined sociodemographic characteristics. Although such associations are not generally expected for germline variants, environmental and genetic factors may interact to influence T2DM susceptibility. Thus, the absence of statistical significance does not exclude possible gene–environment interactions.

Alcohol consumption, higher BMI, and elevated blood pressure were associated with T2DM in this study. The association between increased BMI and T2DM is consistent with established evidence linking excess adiposity to insulin resistance and diabetes [23]. These findings emphasize that genetic susceptibility should be considered alongside established metabolic and environmental factors when investigating T2DM.

Occupation was associated with T2DM, with farmers, daily laborers, and business workers showing lower odds than the reference group. However, this may reflect differences in physical activity, socioeconomic status, diet, obesity, and healthcare access rather than a direct occupational effect. Smoking, khat chewing, and physical activity were not significantly associated with T2DM, although limited sample size, self‐reported data, and residual confounding may have influenced these findings. The non‐significant CC genotype association may also reflect limited statistical power. Larger studies are needed for confirmation.

Biologically, TGF‐β1 may contribute to diabetes through inflammation, extracellular‐matrix regulation, vascular remodeling, and fibrosis. The T869C variant occurs in the signal peptide, suggesting that its effects may involve TGF‐β1 processing or secretion rather than receptor binding. Differences in TGF‐β1 secretion associated with this variant may partly explain its observed association with T2DM [19].

## 5 Conclusion

The study found that occupation, BMI, elevated blood pressure, and alcohol consumption were significantly associated with T2DM. The TGF‐β1 codon 10 T > C polymorphism also differed between cases and controls. The TC genotype (AOR = 3.326, 95% CI: 1.009–10.969, *p* = 0.048) and C allele (AOR = 2.361, 95% CI: 1.231–4.528, *p* = 0.010) were associated with higher odds of T2DM, while the CC genotype was not significant. These findings should be interpreted cautiously and do not establish causality.

## Author Contributions

**Haymanot Getnet:** investigation, validation, formal analysis, resources. **Mequanente Dagnaw:** conceptualization, methodology, investigation, visualization, software, data curation, supervision. **Meera Indracanti:** writing – original draft, methodology, project administration, resources. **Nega Berhane:** conceptualization, funding acquisition, investigation, methodology, software, resources, supervision.

## Funding

The authors have nothing to report.

## Ethics Statement

Ethical approval was obtained from the Institute of Biotechnology IRB (Ref. No. DMB 22/03/2024), followed by permission from Injibara Comprehensive Hospital. The study followed the Declaration of Helsinki.

## Consent

Written informed consent was obtained from all participants. Participation was voluntary, confidentiality was maintained, and participants could withdraw at any time.

## Conflicts of Interest

The authors declare no conflicts of interest.

## Transparency Statement

The corresponding author, Mequanente Dagnaw, affirms that this manuscript is an honest, accurate, and transparent account of the study being reported; that no important aspects of the study have been omitted; and that any discrepancies from the study as planned (and, if relevant, registered) have been explained.

## Supporting information


Supporting File 1

Supporting File 2

Supporting File 3

Supporting File 4

Supporting File 5

Supporting File 6

Supporting File 7

Supporting File 8

Supporting File 9

## Data Availability

The corresponding author will have the right access to the data upon request.
