# Supplementary material for: Molecular Epidemiology of TGF‐β1 Gene Variants and Their Relationship With Adult Type 2 Diabetes Mellitus Patients Visiting Injibara Comprehensive Hospital in Northwest Ethiopia: A Case‐Control Study
Source: Health Sci Rep. 2026 Sep 25;9(10):e73298. doi: 10.1002/hsr2.73298 (PMC13614075; doi:10.1002/hsr2.73298)
Supplement: Supplementary file 1 [file HSR2-9-e73298-s001.docx]

**Supplementary Table 5**. Bivariate and Multivariate analysis of associated behavioral factors with type 2 DM

| Variables | Category | COR (95% CI) | P-Value | AOR (95% CI) | P-value |
| --- | --- | --- | --- | --- | --- |
| Body mass index | Over weight | 3.379(1.555-7.342) | 0.002 | 2.479(1.053-5.838) | .038 |
|  | Normal weight | 1.00 |  | 1.00 |  |
| Blood pressure | Elevated | 4.048(1.882-8.710) | .000 | 3.097(1.345-7.132) | .008 |
|  | Normal | 1.00 |  | 1.00 |  |
| Alcohol consuming | Yes | 4.785(2.240-10.221) | .000 | 4.729(2.084-10.727) | .000 |
|  | No | 1.00 |  | 1.00 |  |
| Smoking habit | Yes | .736(0.247-2.193) | .582 |  |  |
|  | No | 1.00 |  |  |  |
| Chat chewing | Yes | 1.211(0.359-4.086) | .758 |  |  |
|  | No | 1.00 |  |  |  |
| Physical activity | Good | 1.00 |  |  |  |
|  | Poor | 1.000(.589-1.69) | 1.000 |  |  |

*Statically significant: OR: adjusted odd ratio; COR: crudes odd ratio; CI: confidence interval; T2 DM;
