# Supplementary material for: Molecular Epidemiology of TGF‐β1 Gene Variants and Their Relationship With Adult Type 2 Diabetes Mellitus Patients Visiting Injibara Comprehensive Hospital in Northwest Ethiopia: A Case‐Control Study
Source: Health Sci Rep. 2026 Sep 25;9(10):e73298. doi: 10.1002/hsr2.73298 (PMC13614075; doi:10.1002/hsr2.73298)
Supplement: Supplementary file 2 [file HSR2-9-e73298-s002.docx]

**Supplementary Table 7**. Association of TGF-β1 codon 10 T>C and other independent variables

| Variables | | | *TGF-β1* codon 10 T>C  Allelic genotype of Respondents | | | X^2^ Value | P-Value |
| --- | --- | --- | --- | --- | --- | --- | --- |
| Variable | **Category** | **Group** | **TT** | **TC** | **CC** |  |  |
| Body mass index | Normal weight | Case | 35 (30.4%) | 44 (38.3%) | 8 (7.0%) | 1.298 | 0.522 |
|  |  | Control | 71 (61.7%) | 30 (26.1%) | 4 (3.5%) |  |  |
|  | Over weight | Case | 11 (9.6%) | 14 (12.2%) | 3 (2.6%) |  |  |
|  |  | Control | 7 (6.1%) | 2 (1.7%) | 1 (0.9%) |  |  |
| Blood Pressure | Normal | Case | 33 (28.7%) | 43 (37.4%) | 7 (6.1%) | 1.694 | 0.429 |
|  |  | Control | 72 (62.6%) | 28 (24.3%) | 5 (4.3%) |  |  |
|  | Elevated | Case | 13 (11.3%) | 15 (13.0%) | 4 (3.5%) |  |  |
|  |  | Control | 6 (5.2%) | 4 (3.5%) | 0 (0.0%) |  |  |
| Alcohol Consuming | Yes | Case | 30 (26.1%) | 42 (36.5%) | 7 (6.1%) | 1.545 | 0.462 |
|  |  | Control | 69 (60.0%) | 31 (27.0%) | 5 (4.3%) |  |  |
|  | No | Case | 16 (13.9%) | 16 (13.9%) | 4 (3.5%) |  |  |
|  |  | Control | 9 (7.8%) | 1 (0.9%) | 0 (0.0%) |  |  |
