# Supplementary material for: Molecular Epidemiology of TGF‐β1 Gene Variants and Their Relationship With Adult Type 2 Diabetes Mellitus Patients Visiting Injibara Comprehensive Hospital in Northwest Ethiopia: A Case‐Control Study
Source: Health Sci Rep. 2026 Sep 25;9(10):e73298. doi: 10.1002/hsr2.73298 (PMC13614075; doi:10.1002/hsr2.73298)
Supplement: Supplementary file 3 [file HSR2-9-e73298-s003.docx]

**Supplementary Table 2**. Socio-demographic parameters of study participants

| Effects Case | | | | Control | | Total | |
| --- | --- | --- | --- | --- | --- | --- | --- |
| Variable | **Category** | **Freq.(N)** | **Per. (%)** | **Freq.(N)** | **Per. (%)** | **Freq. (N)** | **Per. (%)** |
| Gender | Male | 65 | 56.5 | 54 | 47.0 | 119 | 51.7 |
|  | Female | 50 | 43.5 | 61 | 53.0 | 111 | 48.3 |
| Age | 18-40 | 24 | 20.9 | 26 | 22.6 | 50 | 21.7 |
|  | 41-60 | 71 | 61.7 | 71 | 61.7 | 142 | 61.7 |
|  | >60 | 20 | 17.4 | 18 | 15.7 | 38 | 16.5 |
| Education Status | Non-formal education | 58 | 50.4 | 60 | 52.2 | 118 | 51.3 |
|  | Primary | 22 | 19.1 | 17 | 14.8 | 39 | 17.0 |
|  | Secondary | 24 | 20.9 | 21 | 18.3 | 45 | 19.6 |
|  | Diploma and above | 11 | 9.6 | 17 | 14.8 | 28 | 12.2 |
| Marital Status | Single | 4 | 3.5 | 8 | 7.0 | 12 | 5.2 |
|  | Married | 97 | 84.3 | 87 | 75.7 | 184 | 80.0 |
|  | Windowed | 8 | 7.0 | 9 | 7.8 | 17 | 7.4 |
|  | Separated | 6 | 5.2 | 11 | 9.6 | 17 | 7.4 |
| Place of Residence | Urban | 57 | 49.6 | 61 | 53.0 | 118 | 51.3 |
|  | Rural | 58 | 50.4 | 54 | 47.0 | 112 | 48.7 |
| Occupation Status | Government employee | 9 | 7.8 | 12 | 10.4 | 21 | 9.1 |
|  | Farmer | 41 | 35.7 | 26 | 22.6 | 67 | 29.1 |
|  | Daily laborer | 6 | 5.2 | 2 | 1.7 | 8 | 3.5 |
|  | Businessman dealer | 47 | 40.9 | 51 | 44.3 | 98 | 42.6 |
|  | House wife | 12 | 10.4 | 24 | 20.9 | 36 | 15.7 |
| Religion | Orthodox | 110 | 95.7 | 113 | 98.3 | 223 | 97.0 |
|  | Muslim | 5 | 4.3 | 2 | 1.7 | 7 | 3.0 |
| DM of family history | Yes | 11 | 9.6 |  |  | 11 | 4.8 |
|  | No | 104 | 90.4 | 115 | 100.0 | 219 | 95.2 |
| Family history of hypertension | Yes | 12 | 10.4 |  |  | 12 | 5.2 |
|  | No | 103 | 89.6 | 115 | 100.0 | 218 | 94.8 |
| Average monthly income (ETB) | 1501-3500 | 2 | 1.8 |  |  | 2 | .9 |
|  | 3501-6000 | 25 | 21.7 | 33 | 28.7 | 58 | 25.2 |
|  | >6000 | 88 | 76.5 | 82 | 71.3 | 170 | 73.9 |
|  | Total | 115 | 100.0 | 115 | 100.0 | 230 | 100.0 |
