# Supplementary material for: Molecular Epidemiology of TGF‐β1 Gene Variants and Their Relationship With Adult Type 2 Diabetes Mellitus Patients Visiting Injibara Comprehensive Hospital in Northwest Ethiopia: A Case‐Control Study
Source: Health Sci Rep. 2026 Sep 25;9(10):e73298. doi: 10.1002/hsr2.73298 (PMC13614075; doi:10.1002/hsr2.73298)
Supplement: Supplementary file 4 [file HSR2-9-e73298-s004.docx]

**Supplementary Table 4**. Bivariate and Multivariate analysis of associated sociodemographic factors with type 2 DM

| Variables | Category | COR (95% CI) | P-Value | AOR (95% CI) | P-Value |
| --- | --- | --- | --- | --- | --- |
| Sex | Male | 0.681(.405-1.145) | .147 | .782(0.400-1.530) | .473 |
|  | Female | 1.00 |  | 1.00 |  |
| Age | 18-40 | 1.204(.517-2.801) | .667 |  |  |
|  | 41-60 | 1.111(.543-2.275) | .773 |  |  |
|  | >60 | 1.00 |  |  |  |
| Educational Status | No formal education | 0.669(.289-1.550) | .349 |  |  |
|  | Primary | 0.500(.186-1.343) | .169 | .583(0.183-1.856) | .361 |
|  | Secondary | 0.566(.217-1.476) | .245 |  |  |
|  | Diploma and above | 1.00 |  | 1.00 |  |
| Marital Status | Single | 1.091(0.230-5.185) | .913 |  |  |
|  | Married | 0.489(0.174-1.379) | .176 | .498(0.133-1.860) | .179 |
|  | Windowed | 0.614(0.155-2.432) | .487 |  |  |
|  | Separated | 1.00 |  |  |  |
| Place of Residence | Urban | 1.149(.685-1.928) | .598 |  |  |
|  | Rural | 1.00 |  |  |  |
| Occupation Status | Government employee | .667(.220-2.018) | .473 |  |  |
|  | Farmer | .317(0.136-0.741) | .008 | .090(0.023-0.353) | 0.001 |
|  | Daily laborer | .167(0.029-0.953) | .044 | .026(0.003-0.260) | 0.002 |
|  | Businessman | .543(.244-1.205) | .133 | .188(0.048-0.744) | 0.017 |
|  | House wife | 1:00 |  | 1:00 |  |
| Religion | Orthodox | 1.00 |  |  |  |
|  | Muslim | 2.568(0.488-13.516) | .266 |  |  |
| DM of family history | Yes | .520(0.186-1.458) | .214 |  |  |
|  | No | 1.00 |  |  |  |
| Family history of hypertension | Yes | .556(0.211-1.468) | .236 |  |  |
|  | No | 1.00 |  |  |  |
| Average monthly income (ETB) | 1501-3500 | 2.640(0.447-15.579) | .284 |  |  |
|  | 3501-6000 | 2.640(0.447-15.579) | .480 |  |  |
|  | >6000 | 1.00 |  |  |  |

*Statically significant: OR: adjusted odd ratio; COR: crudes odd ratio; CI: confidence interval; T2 DM;
