# Supplementary material for: Molecular Epidemiology of TGF‐β1 Gene Variants and Their Relationship With Adult Type 2 Diabetes Mellitus Patients Visiting Injibara Comprehensive Hospital in Northwest Ethiopia: A Case‐Control Study
Source: Health Sci Rep. 2026 Sep 25;9(10):e73298. doi: 10.1002/hsr2.73298 (PMC13614075; doi:10.1002/hsr2.73298)
Supplement: Supplementary file 5 [file HSR2-9-e73298-s005.docx]

**Supplementary** **Table 3**. Behavioral and Clinical Parameters of Participants

| Effects Case | | | | Control | | Total | |
| --- | --- | --- | --- | --- | --- | --- | --- |
| **Variable** | **Category** | **Freq.(N)** | **Per.(%)** | **Freq. (N)** | **Per. (%)** | **Freq.(N)** | **Per (%)** |
|  |  |  |  |  |  |  |  |
| Chat chewing | Yes | 5 | 4.3 | 6 | 5.2 | 11 | 4.8 |
|  | No | 110 | 95.7 | 109 | 94.8 | 219 | 95.2 |
| Alcohol consuming | Yes | 105 | 68.7 | 79 | 91.3 | 184 | 80.0 |
|  | No | 10 | 31.3 | 36 | 8.7 | 46 | 20.0 |
| Smoking habit | Yes | 8 | 7.0 | 6 | 5.2 | 14 | 6.1 |
|  | No | 107 | 93.0 | 109 | 94.8 | 216 | 93.9 |
| Physical activity | Good | 70 | 60.9 | 70 | 60.9 | 140 | 60.9 |
|  | Poor | 45 | 39.1 | 45 | 39.1 | 90 | 39.1 |
| BMI | Normal weight | 87 | 75.7 | 105 | 91.3 | 192 | 83.5 |
|  | Over weight | 28 | 24.3 | 10 | 8.7 | 38 | 16.5 |
| Blood pressure | Normal | 83 | 72.2 | 105 | 91.3 | 188 | 81.7 |
|  | Elevated | 32 | 27.8 | 10 | 8.7 | 42 | 18.3 |
| Fasting glucose test | Over | 103 | 89.6 | - | - | 103 | 44.8 |
|  | Normal | 7 | 6.1 | 115 | 100.0 | 122 | 53.0 |
|  | Under | 5 | 4.3 | - | - | 5 | 2.2 |
| DM duration | <one year | 10 | 8.7 | - | - | 10 | 4.3 |
|  | One -to two-year | 34 | 29.6 | - | - | 34 | 14.8 |
|  | >two years | 71 | 60.9 | - | - | 71 | 30.4 |
| Baseline medication | Oral | 84 | 73.0 | - | - | 84 | 36.5 |
|  | Both insulin and oral | 31 | 27.0 | - | - | 31 | 13.5 |
|  | Total | 115 | 100.0 | 115 | 100.0 | 230 | 100 |
