# Supplementary material for: Molecular Epidemiology of TGF‐β1 Gene Variants and Their Relationship With Adult Type 2 Diabetes Mellitus Patients Visiting Injibara Comprehensive Hospital in Northwest Ethiopia: A Case‐Control Study
Source: Health Sci Rep. 2026 Sep 25;9(10):e73298. doi: 10.1002/hsr2.73298 (PMC13614075; doi:10.1002/hsr2.73298)
Supplement: Supplementary file 6 [file HSR2-9-e73298-s006.docx]

**Supplementary Figure 2.** Representative agarose gel electrophoresis showing gene polymorphism products. Lane M contains the 100-bp DNA molecular-weight marker. Lanes 1–4 show representative PCR products with genotypes CC, CC, TT, and TT, respectively. The common PCR product is 465 bp, while the genotype-specific bands are CC = 204 bp and TT = 297 bp. Molecular-weight sizes are indicated on the left side of the gel. The figure was prepared from the original, unprocessed gel image without horizontal flipping, duplication, or rotation.

**Supplementary Figure-2**. Gene polymorphism product on agarose gel electrophoresis documentation
