# Supplementary material for: Molecular Epidemiology of TGF‐β1 Gene Variants and Their Relationship With Adult Type 2 Diabetes Mellitus Patients Visiting Injibara Comprehensive Hospital in Northwest Ethiopia: A Case‐Control Study
Source: Health Sci Rep. 2026 Sep 25;9(10):e73298. doi: 10.1002/hsr2.73298 (PMC13614075; doi:10.1002/hsr2.73298)
Supplement: Supplementary file 7 [file HSR2-9-e73298-s007.docx]

**Supplementary Figure 1. Gene polymorphism products on agarose gel electrophoresis.** Lanes 1–4 show representative genotypes: CC (204 bp), TC (204 bp and 297 bp), TT (297 bp), and CC (204 bp), respectively. The common PCR product is 465 bp. The left and right lanes contain the 100-bp DNA molecular-weight marker used for estimation of PCR product sizes. The sizes of the molecular-weight markers are indicated on both sides of the gel. The figure was prepared from the original gel image without horizontal flipping or rotation.
