# Supplementary material for: Molecular Epidemiology of TGF‐β1 Gene Variants and Their Relationship With Adult Type 2 Diabetes Mellitus Patients Visiting Injibara Comprehensive Hospital in Northwest Ethiopia: A Case‐Control Study
Source: Health Sci Rep. 2026 Sep 25;9(10):e73298. doi: 10.1002/hsr2.73298 (PMC13614075; doi:10.1002/hsr2.73298)
Supplement: Supplementary file 8 [file HSR2-9-e73298-s008.docx]

**Supplementary Table 1**. Primer sequences with melting and annealing temperature

| **SNP** | **Primer sequence (5’ to 3’)** | **Melting Temp** | **Annealing Temp.** | **Products** |
| --- | --- | --- | --- | --- |
| *TGF-β1 (T/C)* | **F1**: 5¢-CTC CAC CAC TGC GCC CTT CT-3¢ | 64℃ | 67℃ | F1 Comman Primer 465 bp |
|  | **R1**: 5¢-AGC AGC GGT AGC AGC AGC G-3¢ (C allele) | 65℃ |  | 204 bp C allele spsfic |
|  | **F2**: 5¢-GGG CTG CGG CTG CTG CT-3¢ (T allele) | 54℃ |  | 297 bp T allele specific |
|  | **R2**: 5¢-GTA GTC GGC CTC AGG CTC GG-3¢ | 62℃ |  | R2 Comman Primer 465bp |
