# Supplementary material for: Molecular Epidemiology of TGF‐β1 Gene Variants and Their Relationship With Adult Type 2 Diabetes Mellitus Patients Visiting Injibara Comprehensive Hospital in Northwest Ethiopia: A Case‐Control Study
Source: Health Sci Rep. 2026 Sep 25;9(10):e73298. doi: 10.1002/hsr2.73298 (PMC13614075; doi:10.1002/hsr2.73298)
Supplement: Supplementary file 9 [file HSR2-9-e73298-s009.docx]

**Supplementary Table 6**. TGF.b1. genotype association: Bivariate and Multivariate analysis

| **Gene** | **Genotype** | **Case** | **Control** | **COR (95%CI)** | **P-value** | **AOR (95%CI)** | **P-Value** |
| --- | --- | --- | --- | --- | --- | --- | --- |
|  | T/T(Reference) | 46 (40%) | 78 (67.8%) | 1.00 |  | 1.00 |  |
|  | T/C | 58 (50.4%) | 32 (27.8%) | 3.730  (1.219-11.412) | .021 | 3.326  (1.009-10.969) | .048 |
| *TGF-b1 T/C* | C/C | 11 (9.6%) | 5  (4.3%) | 1.214  (0.387-3.802) | .739 |  |  |
|  | T | 150  (0.65) | 188  (0.82) | 1.00 |  | 1.00 |  |
|  | C | 80  (0.35) | 42  (0.18) | 2.387  (1.298-4.390) | .005 | 2.361  (1.231-4.528) | .010 |

*Statically significant: OR: adjusted odd ratio; COR: crudes odd ratio; CI: confidence interval;
